# Influencing Factors of Total Skin Irradiation With Helical Tomotherapy

**DOI:** 10.3389/fonc.2022.852345

**Published:** 2022-04-14

**Authors:** Haiyang Wang, Yifei Pi, Yuexin Guo, Xi Pei, Xie George Xu

**Affiliations:** ^1^Institute of Nuclear Medical Physics, University of Science and Technology of China, Hefei, China; ^2^Radiation Oncology Department, The First Affiliated Hospital of Zhengzhou University, Zhengzhou, China; ^3^Technology Development Department, Anhui Wisdom Technology Co., Ltd., Hefei, China; ^4^Radiation Oncology Department, First Affiliated Hospital of University of Science and Technology of China, Hefei, China

**Keywords:** helical tomotherapy, total skin irradiation, influencing factors, auxiliary structure, diving suit

## Abstract

**Purpose:**

To investigate the influencing factors of total skin irradiation (TSI) with helical tomotherapy for guiding the clinical selection of the suitable parameters and optimizing the plan quality and efficiency.

**Materials and Methods:**

Six patients with mycosis fungoides (MF) who received TSI were retrospectively selected. They were all dressed with 5 mm thick diving suits during the CT scan and treatment as a bolus to increase the superficial dose through buildup. The dose prescription was 24 Gy in 20 fractions and 5 times per week. During the planned pretreatment, Ring0, Ring1, Ring2, Ring3, and Ring4 of 1 cm thick away from the planning target volume (PTV) at the distances of 0, 1, 2, 3, and 4 cm and other normal tissues (NTs) were generated, respectively. The auxiliary structures were completely blocked during planning; while the field widths were 5 and 2.5 cm, the pitches were 0.287 and 0.215, the modulation factors were 4 and 3, and the other parameters remained consistent. Finally, the dose parameters of PTV and auxiliary structures, as well as the beam on time (BOT) and gantry period, were compared and analyzed.

**Results:**

when the auxiliary structures were completely blocked with distance to PTV (d_PTV_) above 3 cm were used, the mean dose (D_mean_), conformity index (CI), and heterogeneity index (HI) of the PTV met the clinical requirements. As the d_PTV_ gradually increased, the BOT decreased while the volume of normal tissue that received excessive radiation increased correspondingly. If the d_PTV_ was less than 3 cm, the clinical requirements were not met. The field widths (FWs), pitches, and modulation factors (MFs) had no effect on PTV_mean_ and the HI. The FW of 2.5 cm was slightly better than 5 cm for the CI. The FW and MF had a significant impact on the BOT, which gradually increased with decreasing FW and increasing MF. Pitch had no effect on the BOT.

**Conclusion:**

During planning with TSI patients, d_PTV_ is the key factor that has a significant influence on the plan quality. We found that the plan with the d_PTV_ above 3 cm can meet clinical objectives. The BOT increases as the d_PTV_ increases. The FWs also have an effect on the CI and BOT. Therefore, it is necessary to comprehensively balance these factors to optimize the quality and efficiency of the plan. We also found that different MFs and pitches have no obvious effect on the results.

## Introduction

Cutaneous T-cell lymphoma (CTCL), a relatively rare group of mature T-cell lymphomas, mainly manifests in the skin and accounts for about 71% of all primary cutaneous lymphomas. The common subtype of CTCL is mycosis fungoides (MF) (1). According to the latest statistics data from the National Cancer Institute (NCI) “Monitoring Epidemiology and Results,” CTCL (mainly MF) is currently increasing at a rate of 9.6 cases/million every year, and the incidence rate accounts for about 50% of CTCL (2). CTCL is usually highly radiosensitive, and the traditional treatment technique as well as one of the most effective methods for CTCL is total skin electron irradiation (TSEI) (3). At present, the dual-frame six-field irradiation technology developed by the Stanford University School of Medicine was mostly used; however, the long treatment distance requires the patient to stand and perform multifield irradiation with a rotating gantry, which is a burden for the patient (4). With the development of radiotherapy technology, especially the emergence of helical tomotherapy (HT) (5), which equips 51 equally spaced beam angles at 360-degree helical irradiation, the opening and closing of 64-leaf, pneumatically powered, binary multileaf collimator and translation motion of the treatment couch. These unique components endows HT with many advantages, such as a high degree of freedom, power in dose optimization and the treatment of an ultralong target (160 cm × 40 cm) (6), so HT is very suitable for the treatment of long and complex targets, such as total body multiple metastatic irradiation, craniospinal radiotherapy, total body irradiation, and total marrow irradiation (7). Moreover, compared to the traditional TSEI in which patients are required to stand all the time during treatment, the lying-down treatment method of TSI makes patients less fatigued, more comfortable, and get a better dose distribution. Hsieh CH et al. (8) used a 3 mm diving suit as a bolus. The d_PTV_ was 2.5 cm and the FW, pitch, and MF were 2.5, 0.287, and 3.5 cm; Haraldsson A et al. (9) used a 7 mm diving suit as bolus, and the d_PTV_ was 3 cm as, the FW, pitch and MF was 5, 0.2, and 2.3 cm, respectively, they were designing plans only to meet clinical objectives without a further study of the optimal planning and the effect of other parameters on the results. The purpose of this work is to study the influence of the different parameters and auxiliary structures of the complete mode on the plan quality, in order to guide the clinical selection of the best parameters combination and improve the plan quality and performance.

## Materials and Methods

### Patients’ Clinical Characteristics

The first six consecutive patients with MF who received TSI between 2020 and 2021 at the Department of Radiotherapy of the First Affiliated Hospital of Zhengzhou University were retrospectively selected, as all patients had pathologically confirmed.

### Bolus

Six patients were dressed in the 5 mm diving suit as a bolus. The diving suits were tailored according to the patients’ external shape to achieve a tight wrap around the body.

### Immobilization

**Figure 1:**
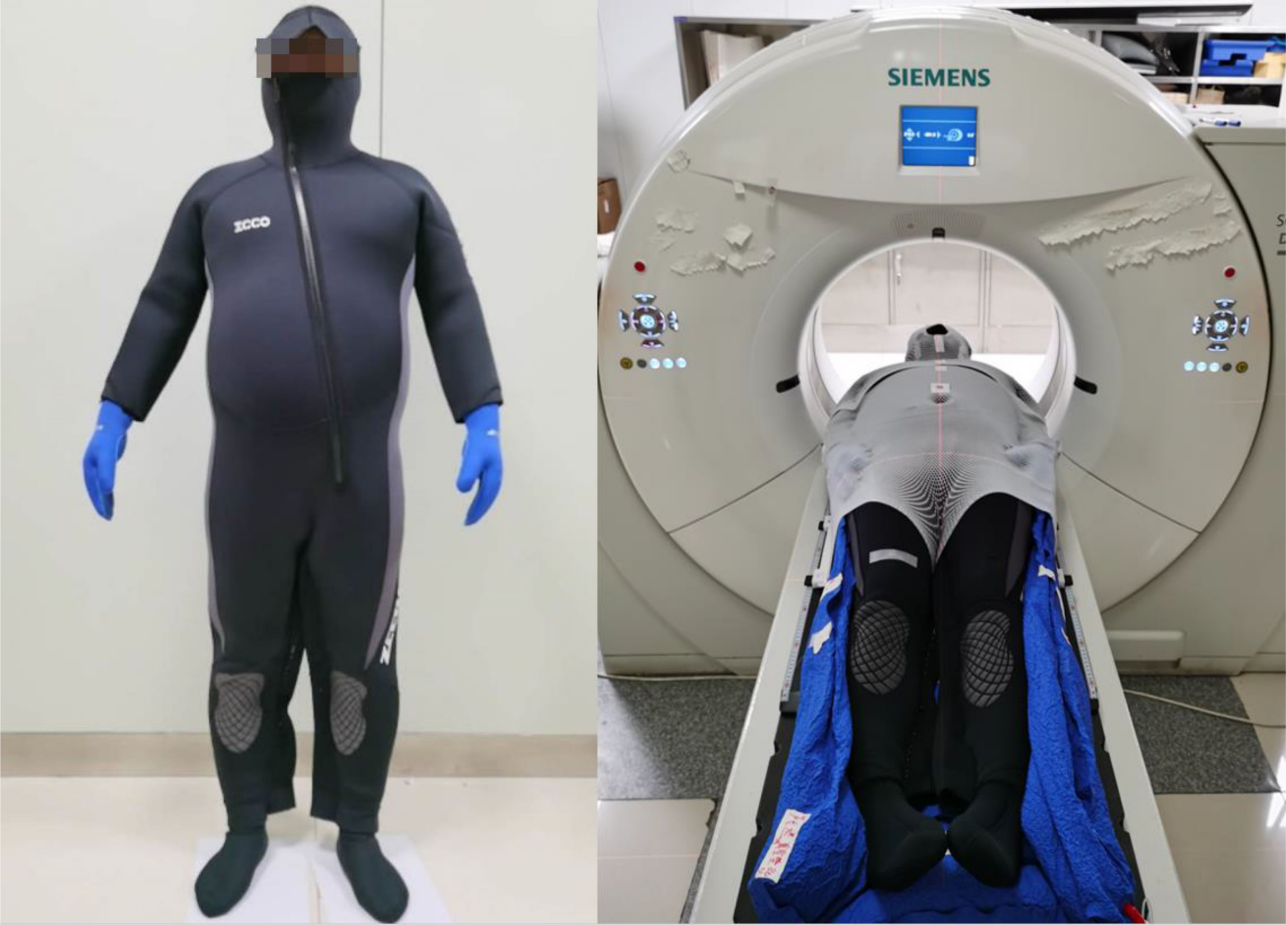
Patient dressed with a 5 mm diving suit.

Patients dressed with a 5 mm diving suit were immobilized in a supine position (**Figure 1**). Thermoplastic masks were used for the head and neck, thorax, and abdomen, while the lower limbs were immobilized in a vacuum cushion. The upper marks and lower masks are located near the patient’s belly button and near the patient’s patella, respectively, and the segment line made of lead is located around 10 cm above the patella as the boundary between the upper and lower target.

### Image Acquisition at Simulation

Helical computed tomography (CT) scans (SOMATOM Definition AS40; Siemens) were performed under the following condition: a scan and reconstruction slice thickness of 5 mm. The patients were scanned in upper and lower segment, respectively. The upper segments were scanned from the skull to 10 cm below the boundary, while the lower segments scanned from the toes to 10 cm above the boundary.

### Delineation of Target Volumes and Organs at Risk

The target volumes and organs at risk **(**OARs) for all patients were delineated by radiation oncologists based on the planning CT according to the ICRU50 (10) and ICRU62 reports (11). The clinical target volume (CTV) was defined between the skin surface and 5 mm below it (8). The planning target volume (PTV) was generated by expanding the CTV with 5 mm and then retracting it with 3 mm in the outside region, considering the setup error and dose buildup effect. OARs were delineated based on the ICRU 83 report (12), primarily including the total bone marrow (head and neck bones, upper limb bones, ribs, spine, pelvis, lower limb bones), eyeballs, lens, parotid, lungs, heart, kidneys, liver, bladder, rectum, spinal cord, and brainstem. The junction between the upper and lower sections of TBI had been studied in our previous publication (13); the dose in the overlap region was mostly homogeneous when the distance was equal to the FW.

### Plan Designs

**Figure 2:**
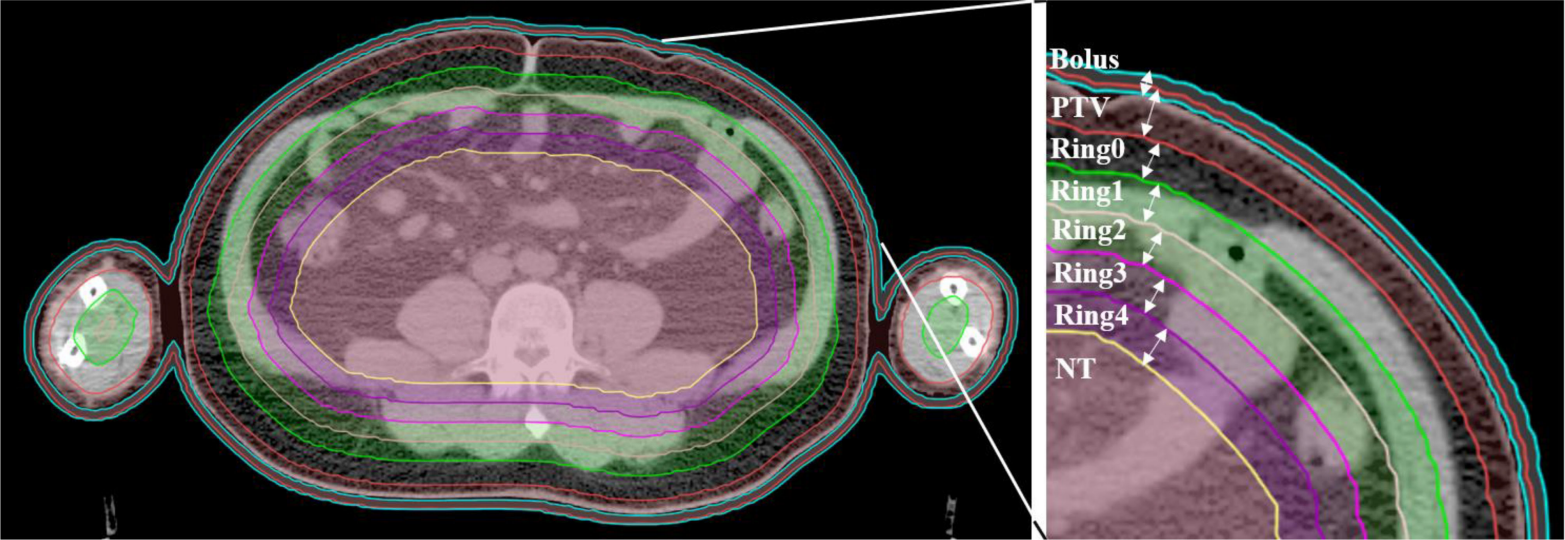
Structure sketches of the auxiliary structure Ring0, Ring1, Ring2, Ring3, Ring4, and NT as well as the PTV and bolus (the diving suit).

**Figure 3:**
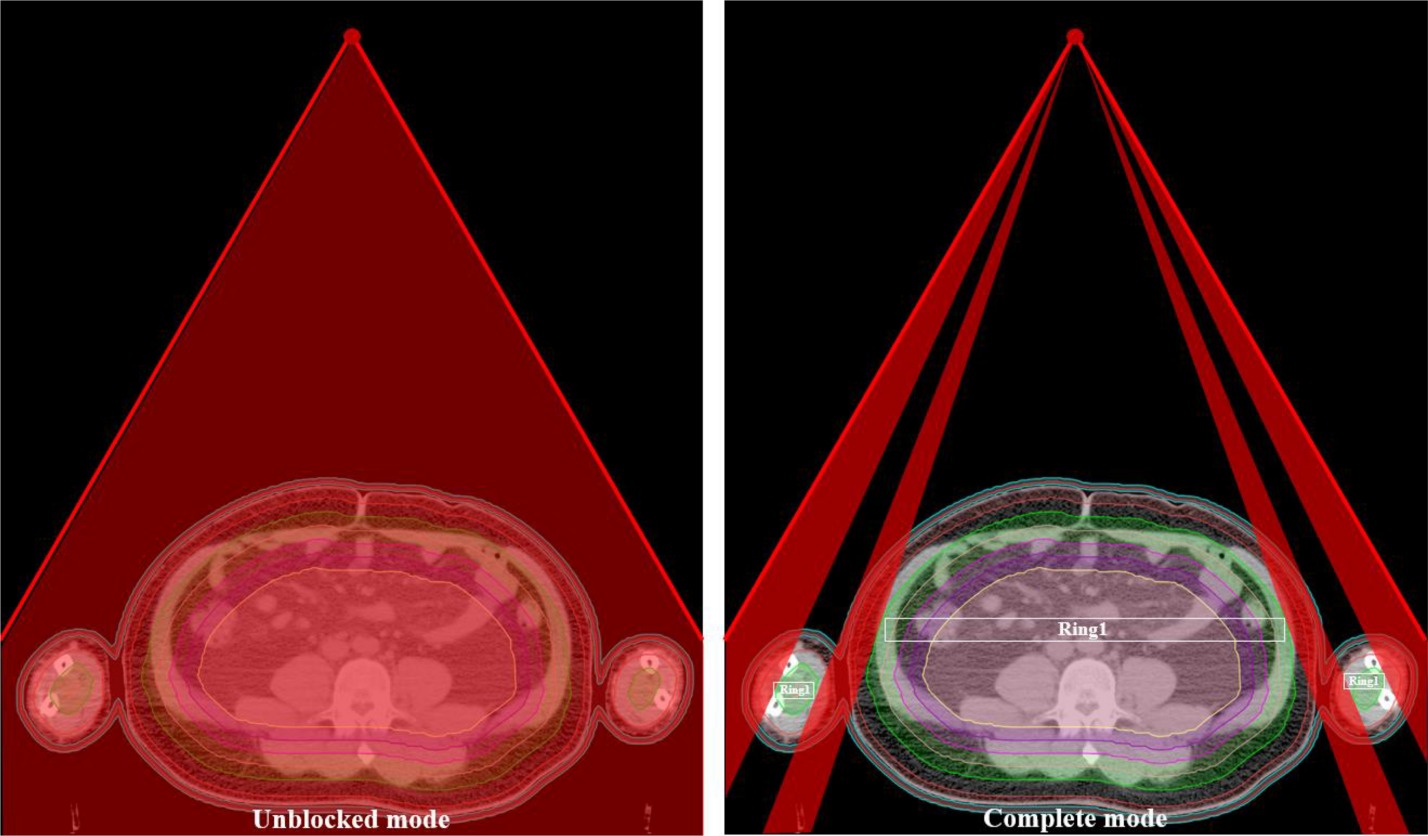
The ray distribution of the unblocked mode and complete mode of the Ring1.

The planning CT and contoured structures of each patient were transferred to the treatment planning workstation (Version 5.1.6; Accuray, Sunnyvale, CA, USA) for planning. The dose prescription was 24 Gy in 20 fractions and 5 times per week. The PTV gradually retracted from 1 to 5 cm by a 1 cm step to create the ring-shape auxiliary structure as Ring0, Ring1, Ring2, Ring3, and Ring4 and the solid NT auxiliary structures as NT (**Figure 2**). The auxiliary structures were used as an assistant tool for plan optimization to achieve the dose objectives. During planning, all the auxiliary structures were set to a complete mode one by one (**Figure 3**) with the FW of 5 and 2.5 cm, pitches of 0.287 and 0.215, and MF of 4 and 3. The dose grid was 0.195 cm × 0.195 cm. Plans were designed by combining different parameters and auxiliary structures; the other parameters remained consistent. Since the planning quality did not improve significantly more than 100 iterations, the final dose calculation was performed after 100 iterations for each plan.

### Assessment of Plan Parameters

The parameters assessed for the patients included the mean dose, heterogeneity index (HI), and conformity index (CI) of the target volume. At least 95% of the target volumes reached the prescribed dose. The HI was calculated using the formula, HI=D_5%_/D_95%_, D_5%_ is the dose received by 5% of the PTV volume, D_95%_ is the dose received by 95% of the volume of the PTV. The HI value greater than 1 represents the heterogeneity dose distribution of the target volume. The CI was obtained using the following Paddick equation (14): CI=V_T,ref_/V_T_ × V_T,ref_/V_ref,_ V_T,ref_ is the target volume covered by the prescription isodose (cm^3^), V_ref_ is the volume encompassed by the prescription isodose (cm^3^), and *V_T_* is the target volume (cm^3^). The CI value is closer to 1, the better dose conformity of the target volume.

### Statistical Analysis

All statistical analyses were performed using SPSS version 19.0 for Windows (IBM Corp., Armonk, NY, United States). The data results were expressed as mean ± standard deviation (x ± s). The graphics were plotted by Origin version 8.0 for Windows (OriginLab Corp., Northampton, MA, United States).

## Results

### Patients’ Characteristics Comparisons of Dosimetric Parameters of Target Volumes

**Figure 4:**
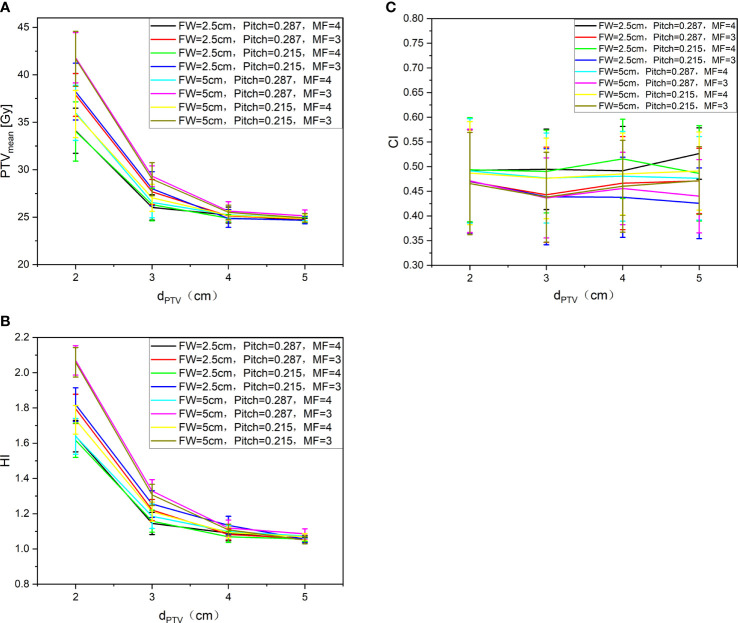
PTVmean **(A)**, HI **(B)**, and CI **(C)** with different auxiliary structures completely blocked.

All the treatment plans met the requirement of 95% prescribed dose coverage of the target volume. **Figure 4** shows the trend of the PTV_mean_ (A), HI (B), and CI (C) under different auxiliary structures (Ring2, Ring3, Ring4, NT) of the complete mode with FW=5 cm/2.5 cm, Pitch=0.287/0.215, MF=4/3. It can be clearly observed from **Figure 4** PTV_mean_ (A): with the d_PTV_ increasing, the mean dose to PTV gradually approaches the prescribed dose. When the d_PTV_ is above 3 cm, the mean dose to PTV meets the clinical requirements that reach the prescribed dose; FW, Pitch, and MF had no effect on the results. **Figure 4** HI (B) shows the result: when the d_PTV_ is above 3 cm, the HI of the target can be better; the result was consistent with PTV_mean_. **Figure 4** CI (C) shows that the auxiliary structures have no obvious influence on the CI of the target. Relatively speaking, an FW of 2.5 cm was slightly better than 5 cm, but Pitch and MF had no effect on the CI. The most important influencing factor on the dose distribution of the target was the auxiliary structure of the complete mode; the recommended distance was greater than or equal to 3 cm, while FW, Pitch, and MF have no significant effect on the results. A total of 6 patients with MF received TSI; 4 patients are men and 2 patients are women as shown in the table. The age range and average are 31–65 and 51, the height range and average are 155–172 cm and 165 cm, and the weight range and average are 40–95 kg and 64.7 kg. Detailed information were listed in **Table 1**.

**Table 1:** 6 patients’ characteristics.

Patient no	Age (years)	Sex	Diagnosis	Body length (cm)	Body weight (kg)	Treatment technique
1	31	M	MF	170	95	TSI
2	65	F	MF	155	40	TSI
3	42	M	MF	165	65	TSI
4	56	M	MF	172	74	TSI
5	52	F	MF	160	51	TSI
6	60	M	MF	168	63	TSI

### Comparisons of Dosimetric Parameters of Auxiliary Structure

**Figure 5:**
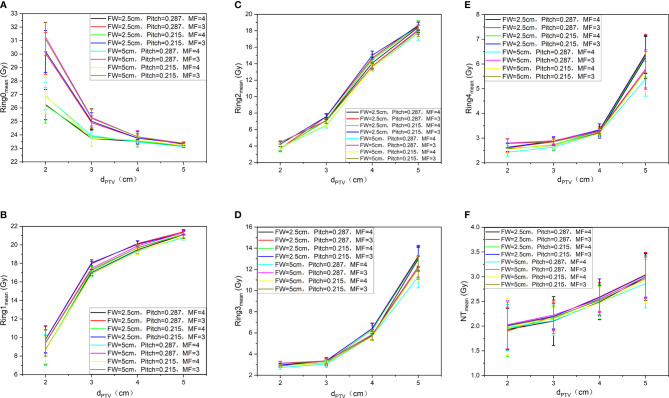
Trend of the mean dose of Ring0 **(A)**, Ring1 **(B)**, Ring2 **(C)**, Ring3 **(D)**, Ring4 **(E)**, and NT **(F)** with auxiliary structures (Ring2, Ring3, Ring4, NT) of the complete mode, FW=5 cm/2.5 cm, Pitch=0.287/0.215, and MF=4/3.

**Figure 5** shows the trend of the mean dose of Ring0 (A)、Ring1 (B)、Ring2 (C)、Ring3 (D)、Ring4 (E)、and NT (F) with auxiliary structures (Ring2, Ring3, Ring4, NT) of the complete mode, FW=5 cm/2.5 cm, Pitch=0.287/0.215, and MF=4/3. We can observe from **Figure 5** that to ensure that the mean dose of the auxiliary structure Ring0, Ring1, Ring2, Ring3, Ring4, and NT reaches the prescribed dose, the d_PTV_ must be greater than or equal to 3 cm. The figure Ring0 (A)、Ring1 (B)、and Ring2 (C) show that the goal auxiliary structures Ring0, Ring1, and Ring2 meet the above conditions, and the mean dose can reach the prescribed dose. In figures Ring3 (D)、Ring4 (E)、and NT (F), none of Ring3, Ring4, and NT meet the above conditions, and the mean dose is unable to reach the prescribed dose. This conclusion is consistent with results of the target, and FW, Pitch, and MF have no significant influence on the results.

### Comparisons of Beams on Times and Gantry Periods

**Figure 6:**
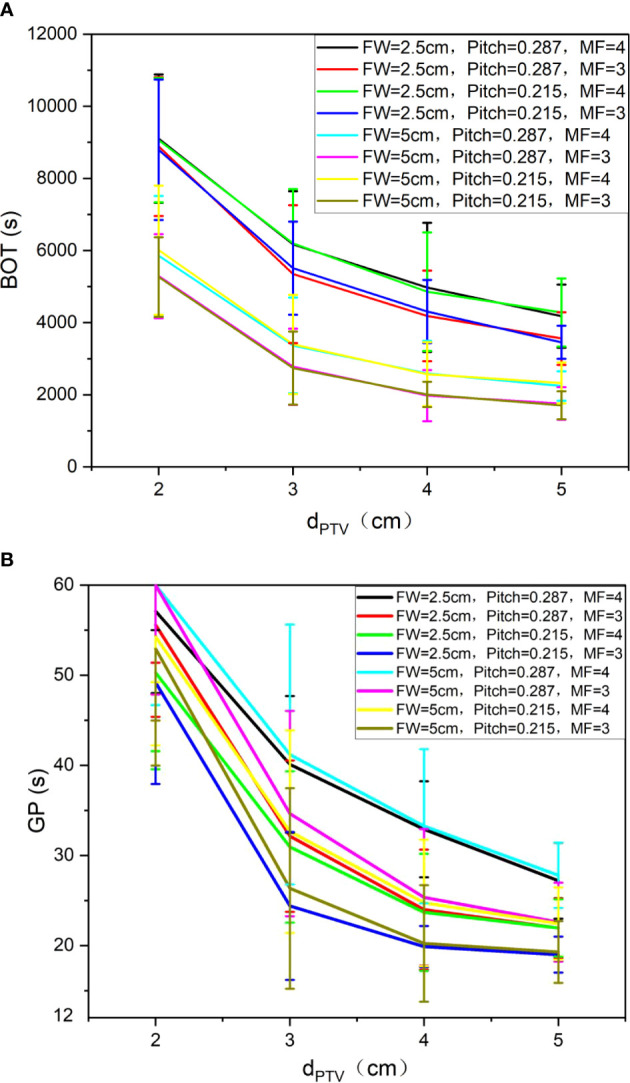
Trend of BOT **(A)** and GP **(B)** under different auxiliary structures (Ring2, Ring3, Ring4, NT) of the complete mode, FW=5. cm/2.5 cm, Pitch=0.287/0.215, and MF=4/3.

**Figure 6** shows the trend of BOT (A) and GP (B) under different auxiliary structures (Ring2, Ring3, Ring4, NT) of the complete mode, FW=5 cm/2.5 cm, Pitch=0.287/0.215, and MF=4/3. We can observe that as the d_PTV_ increases, BOT and GP are gradually reduced. When the d_PTV_ is greater than or equal to 3 cm, the changing tends of BOT (A) and GP (B) are gentle; compared with 5 cm, the BOT for FW of 2.5 cm increased 86 ± 19%, but different MF and Pitch have no significant effect on the results.

In summary, when designing a TSI plan with helical tomotherapy, the most influential factor is the selection of the auxiliary structure in the complete mode. Only the d_PTV_ above 3 cm can meet the clinical requirements. The BOT decreases with the gradual increasing of the d_PTV_. The FW has effect on the CI of the target and the BOT. Therefore, it is necessary to comprehensively balance these factors to optimize the quality and efficiency of the plan. We also concluded that different modulation factors and pitches have no obvious influence on the results.

## Discussion

So far, many medical institutes have treated TSI patients with HT. Due to the lack of treatment guidelines and standards, the auxiliary structures of the complete mode and planning parameters determined by each institute for planning are quite different, which have a significant impact on the plan quality and efficiency. Hsieh CH et al. (8) were the first to achieve TSI with HT, a d_PTV_ of 2.5 cm, an FW of 2.5 cm, Pitch of 0.287, and MF of 3.5 were used in their study. However, Haraldsson A et al. (9) selected a d_PTV_ of 3 cm, with an FW of 5 cm, Pitch of 0.2, and MF of 2.3 for planning. Geurts M et al. (15)selected a d_PTV_ of 5 cm, an FW of 5 cm, Pitch of 0.215, and MF of 3. Sarfehnia A et al. (16) selected a d_PTV_ of 2 cm, FW of 5 cm, Pitch of 0.287, and MF of 2.5. Lin CT et al. (17) selected a d_PTV_ of 1.8 cm on the phantom with an FW of 2.5 cm, Pitch of 0.287, and MF of 3.5. Geurts M et al. (15) selected a d_PTV_ of 5 cm, with an FW of 5 cm, Pitch of 0.215, and MF of 3. Different institutes choose various parameters to design a TSI plan with HT in order to reduce the influence of human factors on the plan quality; the various auxiliary structures of the complete mode and planning parameters are studied one by one in this study to obtain the best combination between the auxiliary structures of the complete mode and planning parameters and forming treatment guidelines and standards.

All patients were dressed with a 5 mm diving suit as the bolus in this study because the 5 mm diving suit is the most common one in the market as the bolus; it is easy to obtain, and the research results will be more universal. Mainly considering the low density of the diving suit (-400~-600HU), the impact of dose is relatively small within a relatively small thickness variation range (such as within 1 mm), and the conclusion can be used for reference. However, for the diving suit of different materials (especially with large density differences) as the bolus, more work is required for a robust conclusion.

The PTV gradually retracts into the body to form 1 cm thick auxiliary structures; the volume from the distance 0, 1, 2, 3, and 4 cm and the remaining volume from the PTV are sequentially generated as the auxiliary structures of Ring0, Ring1, Ring2, Ring3, Ring4, and NT with a thickness of 1 cm. The auxiliary structures do not represent OARs or the PTV; they are only used as a tool for plan optimization to achieve dose constraints. At the same time, it is also used as a part of the plan evaluation to study the trend of the dose distribution of the auxiliary structures with distance. The auxiliary structures are formed by the stepwise movement distance of PTV into the body, 0, 1, 2, 3, and 4 cm and the remaining volume from the target sequentially are generated as Ring0, Ring1, Ring2, Ring3, Ring4, and NT auxiliary structures with a thickness of 1 cm. These structures are automatically generated by the physician workstation according to specific requirements, and will not be affected by human factors, so the research results and clinical applications are more universal and representative.

In this study, the auxiliary structures of Ring0, Ring1, Ring2, Ring3, and Ring4 with a thickness of 1 cm are generated and the remaining normal tissue volume is defined as NT. Only 5 auxiliary structures are generated, and no more auxiliary structures are made in this work; because the minimum cross-section of the head and neck is approximately 10 cm, more auxiliary structures are not able to be generated. At the same time, the maximum distance of auxiliary structures used in related research is 5 cm according to the literature (15). The auxiliary structures selected are 1 cm thick in this study, and no thinner auxiliary structures (such as 8, 5, and 3 mm) are generated. The main consideration is that the research results with thinner auxiliary structures are possibly more refined compared with 1 cm, but the change trend of the results should be consistent.

This study only contains data for Ring2, Ring3, Ring4, and NT without Ring0 and Ring1. The main reason is that the distance from the PTV is too small to optimize the plan when the Ring0 and Ring1 auxiliary structures are used in the complete mode, so no statistical results are available for Ring0 and Ring1.

This work studies the impact of auxiliary structures and planning parameters on the results. The analysis of the results employs the auxiliary structures of Ring0, Ring1, Ring2, Ring3, Ring4, and NT for evaluation instead of using the actual OARs for statistics. The analysis mainly investigated six patients who have large differences in height, weight, and weight, these factors may have a great impact on OARs. For example, the larger weight means the more fat under the skin can protect the OARs better. The lighter weight is unable to protect OARs due to the less fat under the skin; therefore, it is not universal. The only variable of the auxiliary structures selected in this work is the d_PTV_, which means that fewer influencing factors could disturb the results and this work can well reflect the dose drop gradient of the body and is more universal and representative.

In order to study the influence of various parameters on the results, this work chooses FW=5 cm/2.5 cm, Pitch=0.215/0.287, MF=3/4, mainly from clinical reports and related research literature as references. The FWs of 5 and 2.5 cm are defined based on the execution efficiency; The pitches of 0.215 and 0.287 are applied considering the GP; The MFs of 3 and 4 are used based on the actual complexity of the plan. Calculation grid affects were studied with one patient (d_PTV_=4 cm, FW=5 cm, MF=3, pitch=0.287), the results showed that PTV_mean_ was decreased (26.75 ± 1.47 Gy, 26.54 ± 1.43 Gy, 20.30 ± 1.41 Gy), but GPs (29.29, 29.70, and 30.20 s) and BOTs (2,364.10, 2,395.20, and 2,434.90 s) increased from fine to coarse calculation resolution. Considering that the calculation grid affects the accuracy of dose calculation, this work adopts the finest grid of 0.195 cm × 0.195 cm. During the experiment, only one variable was changed at a time, and the other variables remained unchanged. The dose calculation for the plan is performed after 100 iterations to ensure the consistency and repeatability of the results.

Haraldsson A et al. (9) used a phantom for the robustness of plan by adding an 8 mm virtual bolus outside the skin for planning optimization; the impact of the positioning error within the 10 mm range on the treatment accuracy is eliminated. Geurts M et al. (15) used an anthropomorphic phantom and added a separate “flash” rind by expanding the external contour by 1.0 cm. The density of the flash structure was overridden to 0.2 g/cm^3^, and a dose constraint equal to the target was chosen. The plan quality began to significantly degrade if the phantom was offset by more than 10 mm using 7.0 and 10 mm target and flash thicknesses. Takenaka R et al. (18) performed total scalp irradiation, a virtual bolus with a thickness of 8 mm and a density of 0.2 g/cm^3^ is added to the total scalp to increase the skin dose, which is the ability to avoid dose hotspots on the scalp and eliminate the impact of positioning errors within 6 mm on the treatment accuracy. Moliner G (19) et al. studied how to optimize the selection of the density and thickness for a virtual bolus when performing TSI with HT to ensure the accuracy and stability of the radiation. Through the comparative study of the four groups of different physical densities of, 0.2, 0.4, and 1 g/cm^3^ and the four groups of a virtual bolus with different thicknesses of 5, 10, 5 + 3, and 10 + 3 mm, they found that a virtual bolus made of a physical density of 0.4 g/cm^3^ and a thickness of 8 mm can eliminate the impact of positioning errors within the range of 2.9 cm on treatment accuracy.

A hematopoietic bone marrow is very radiation sensitive and it can be considered the most important OAR for TSI. Buglione et al. (20) compared the whole bone marrow TSI plan of V_10Gy_, V_12Gy_, and V_20Gy_ values ranging respectively between 23% and 43%, 20.1 and 38% and 9.8 and 24% with TSEBI plan V_10Gy_, V_12Gy_,and V_20Gy_ values ranging respectively between 6.6% and 17%, 5.1% and 14.5%, and 2.8% and 9.6%. Compared with TSI, TSEBI has advantages in terms of toxicity and is generally the first choice for treatment. However, TSI should be considered for large, convex, cutaneous areas, and special requirements for less fatigue, more comfortable and better dose distribution.

The irradiation range of TSI is wide, and the irradiated organs cover almost all parts of the body. The steep dose distribution is great significance to protect normal tissues. Using an auxiliary ring to limit the dose is one of the ways. The single influence of rings to achieve a better dose drop was studied. The bone marrow as a difficult-to-reach condition will seriously affect dose distributions, which is not conducive to the analysis of variables. To achieve the effect of control variables, we only used rings as optimization, ignoring bone marrow conditions. The distribution of the TSI radiation dose under the combined effect of the bone marrow and ring is the focus of the next step.

## Conclusion

The conclusion of this study has been applied in the follow-up treatment of patients in the department. It not only significantly reduces the patient’s normal tissue dose but also meets the clinical prescription requirements. Moreover, the differences caused by human factors during planning between the planner are eliminated, and the overall plan quality and efficiency are improved.

## Data Availability Statement

The raw data supporting the conclusions of this article will be made available by the authors, without undue reservation.

## Author Contributions

Paper idea: HW, YP, YG, XP, and XX. The source of datasets: HW, YP, and XP. Writing of the paper: HW, YP, YG, XP, XX. All authors contributed to the article and approved the submitted version.

## Funding

Natural Science Foundation of Anhui Province, Grant/Award Number: 1908085MA27 China International Medical Foundation Tumor Precision Radiotherapy Spark Program Clinical Research Fund (HDRS2020010110).

## Conflict of Interest

Author XP was employed by Anhui Wisdom Technology Co.

The remaining authors declare that the research was conducted in the absence of any commercial or financial relationships that could be construed as a potential conflict of interest.

## Publisher’s Note

All claims expressed in this article are solely those of the authors and do not necessarily represent those of their affiliated organizations, or those of the publisher, the editors and the reviewers. Any product that may be evaluated in this article, or claim that may be made by its manufacturer, is not guaranteed or endorsed by the publisher.

## References

[1] Willemze R Jaffe ES Burg G Cerroni L Berti E Swerdlow SH. WHO-EORTC Classification for Cutaneous Lymphomas. Blood (2005) 105(10):3768–85. doi: 10.1182/blood-2004-09-3502 15692063

[2] Yu JB Blitzblau RC Decker RH Housman DM Wilson LD. Surveillance, Epidemiology, and End Results (SEER) Database Analysis of Stage IE Primary CD30+ Cutaneous T-Cell Lymphoma (PCCTCL). Int J Radiat Oncol Biol Phys (2007) 69(3):S536–7. doi: 10.1016/j.ijrobp.20

[3] Van Vloten WA De Vroome H Noordijk EM. Total Skin Electron Beam Irradiation for Cutaneous T-Cell Lymphoma (Mycosis Fungoides). Br J Dermatol (1985) 112(6):697–702. doi: 10.1111/j.1365-2133.1985.tb02340.x 3924088

[4] Piotrowski P Milecki M Skórska D Fundowicz D. Fundowicz Total Skin Electron Irradiation Techniques: A Review. Postep Derm Alergol (2013) 30(1):50–5. doi: 10.5114/pdia.2013.33379 PMC3834692 24278046

[5] Mackie TR Balog J Ruchala K Shepard D Aldridge S Fitchard E. Tomotherap. Semin Radiat Oncol (1999) 9(1):108–17. doi: 10.1016/s1053-4296(99)80058-7 10196402

[6] Mackie TR. History of Tomotherapy. Phys Med Biol (2006) 51:R427–53. doi: 10.1088/0031-9155/51/13/R24 16790916

[7] Sterzing F Schubert K Sroka-Perez G Kalz J Debus J Herfarth K. Helical Tomotherapy. Strahlenther und Onkol (2008) 184(1):8–14. doi: 10.1007/s00066-008-1778-6 18188517

[8] Hsieh CH Shueng PW Lin SC Tien HJ Shiau AS Chou YH. Helical Irradiation of the Total Skin With Dose Painting to Replace Total Skin Electron Beam Therapy for Therapy-Refractory Cutaneous CD4+ T-Cell Lymphoma. BioMed Res Int (2013) 2013:1–11. doi: 10.1155/2013/717589 PMC3794623 24175298

[9] Haraldsson A Engleson J Bäck S ÅJ Engelholm S Engström PE. A Helical Tomotherapy as a Robust Low-Dose Treatment Alternative for Total Skin Irradiation. J Appl Clin Med Phys (2019) 20(5):44–54. doi: 10.1002/acm2.12579 31033159 PMC6522990

[10] ICRU. Prescribing, Recording, and Reporting Photon Beam Therapy. ICRU Report 50. Bethesda, MD: International Commission on Radiation Units and Measurements (1993). doi: 10.1118/1.597396

[11] Wambersie A Landberg T. ICRU Report 62, Prescribing, Recording and Reporting Photon Beam Therapy (Supplement to ICRU Report 50). ICRU News (1999) 32:1–155. doi: 10.1093/jicru/os32.1.Report62

[12] International Commission on Radiation Units and Measurements. Report83. Prescribing, Recording, and Reporting Photon- Beam Intensity-Modulated Radiation Therapy (IMRT). Oxford: Pergamon Press (2010). doi: 10.1016/j.canrad.2011.04.003

[13] Wang HY Liu JQ Pi YF Liu Q Mi Y Yang XX. Factors Affecting Dose Distribution in the Overlap Region of Two-Segment Total Body Irradiation by Helical Tomotherapy. Radiat Oncol (2020) 15(1):1–7. doi: 10.1186/s13014-020-01698-x PMC7648982 33160374

[14] Singh VP Kansal S Vaishya S Julka PK Mehta VS. Early Complications Following Gamma Knife Radiosurgery for Intracranial Meningiomas. J Neurosurg (2000) 93(supplement_3):57–61. doi: 10.3171/jns.2000.93.supplement_3.0057 11143263

[15] Geurts M Bayliss A Thapa B. TomoTherapy Total Skin Treatment for Mycosis Fungoides[DB/OL] (2014). Available at: https://www.researchgate.net/publication/267453606_Total_Skin_TomoTherapy_for_Treatment_of_Mycosis_Fungoides.

[16] Sarfehnia A Poon E Davis SD Fleming A Mitchell D Freeman CR. A Novel Approach to Total Skin Irradiation Using Helical TomoTherapy [J]. Pract Radiat Oncol (2014) 4(5):330–5. doi: 10.1016/j.prro.2013.10.004 25194102

[17] Lin CT Shiau AC Tien HJ Yeh HP Shueng PW Hsieh CH. An Attempted Substitute Study of Total Skin Electron Therapy Technique by Using Helical Photon Tomotherapy With Helical Irradiation of the Total Skin Treatment: A Phantom Result. BioMed Res Int (2013) 2013:1–7. doi: 10.1155/2013/108794 PMC3747477 23984313

[18] Takenaka R Haga A Nawa K Hideomi Y Nakagawa K. Improvement of the Robustness to Set Up Error by a Virtual Bolus in Total Scalp Irradiation With Helical TomoTherapy. Radiol Phys Technol (2019) 12(4):433–7. doi: 10.1007/s12194-019-00539-1 31642033

[19] Moliner G Izar F Ferrand R Bardies M Ken S Simon L. Virtual Bolus for Total Body Irradiation Treated With Helical Tomotherapy. J Appl Clin Med Phys (2015) 16(6):164–76. doi: 10.1120/jacmp.v16i6.5580 PMC5691005 26699568

[20] Buglione M Spiazzi L Urpis M Baushi L Avitabile R Pasinetti N. Light and Shadows of a New Technique: Is Photon Total-Skin Irradiation Using Helical IMRT Feasible, Less Complex and as Toxic as the Electrons One? Radiat Oncol (2018) 13(1):158–70. doi: 10.1186/s13014-018-1100-4 PMC6114532 30157892

